# Comparison of visual outcomes of a diffractive trifocal intraocular lens and a refractive bifocal intraocular lens in eyes with axial myopia: a prospective cohort study

**DOI:** 10.1186/s12886-022-02626-1

**Published:** 2022-10-20

**Authors:** Tong Sun, Yiyun Liu, Yufei Gao, Chuhao Tang, Qianqian Lan, Tingting Yang, Xiaorui Zhao, Hong Qi

**Affiliations:** 1 Department of Ophthalmology, Peking University Third Hospital, Beijing, China; 2 Beijing Key Laboratory of Restoration of Damaged Ocular Nerve, Peking University Third Hospital, Beijing, China; 3 Department of Ophthalmology, Affiliated Hospital of Yunnan University, Kunming, China; 4 Department of Ophthalmology, the People’s Hospital of Guangxi Zhuang Autonomous Region, Nanning, China; 5 Present Address: Beijing, P R China

**Keywords:** Cataract, Multifocal intraocular lens, Axial length, Myopia, Visual quality

## Abstract

**Background:**

To assess and compare the efficacy, safety, accuracy, predictability and visual quality of a diffractive trifocal intraocular lens (IOL) and a refractive rotationally asymmetric bifocal IOL in eyes with axial myopia.

**Methods:**

This prospective cohort study enrolled patients with implantation of the diffractive trifocal IOL or the refractive bifocal IOL. Eyes were divided into four groups according to the IOL implanted and axial length. Manifest refraction, uncorrected and corrected visual acuity at far, intermediate and near distances, prediction error of spherical equivalent (SE), contrast sensitivity and aberrations were evaluated three months after surgery.

**Results:**

In total, 80 eyes of 80 patients were included: 20 eyes in each group. Three months postoperatively, the corrected distance visual acuity of two trifocal groups were significantly better than the axial myopia bifocal group (*P* = 0.007 and 0.043). There was no significant difference of postoperative SE (*P* = 0.478), but the SE predictability of the trifocal IOL was better, whether in axial myopia groups (*P* = 0.015) or in control groups (*P* = 0.027). The contrast sensitivity was similar among four groups. The total aberration, higher order aberration and trefoil aberration of bifocal groups were significantly higher (all *P* < 0.001).

**Conclusions:**

The diffractive trifocal IOL and the refractive bifocal IOL both provided good efficacy, accuracy, predictability and safety for eyes with axial myopia. By contrast, the trifocal IOL had a better performance in corrected distance visual acuity and visual quality.

**Trial registration:**

The study was retrospectively registered and posted on clinicaltrials.gov at 12/02/2020 (NCT04265846).

## Introduction

With the development of technology and medical equipment, today’s cataract surgery is able to provide patients with good visual acuity at different distances to prevent presbyopia [1]. Multifocal intraocular lenses (MIOLs), aimed to meet the increasing demand for spectacle independence, has played a very important role in cataract surgery [2]. AT LISA tri 839MP (Carl Zeiss Meditec, Jena, Germany), a diffractive trifocal intraocular lens (IOL) which distributes the light into 3 focal points, has already been proved to be able to provide great visual quality while correcting presbyopia [3–6]. SBL-3 (Lenstec, Inc., Christ Church, Barbados), a refractive rotationally asymmetric bifocal IOL, has also been widely adopted by surgeons around the world [7–9].

In the worldwide, almost 26.5% of adults and 11.7% of adolescents are suffering from myopia [10]. In southern China, approximately 78.4% of adolescents are suffering from myopia, and this proportion has reached 80% in northern China [11, 12]. Unfortunately, with the more and more widespread use of computers and smart phones, these ratios tend to rise gradually. In the future, the proportion of myopia in patients undergoing cataract surgery will also gradually increase. In addition, there seems to be a connection between high myopia and cataract formation. Previous studies have shown that people with spherical equivalent less than -0.5 diopter (D) have a 2–5 times higher risk of developing nuclear cataract and a 30 percent higher risk of developing posterior subcapsular cataract [13, 14]. However, patients with myopia have a susceptibility to other eye diseases, such as retinal diseases and strabismus. It will also add difficulty to the calculation of the required IOL power for cataract patients [15–18]. Despite these difficulties, several studies have applied MIOLs in the treatment of cataract in patients with myopia, which has been proved to be effective [19, 20].

In this study, we analyzed postoperative visual acuity at different distances, postoperative spherical equivalent (SE) and its prediction error, contrast sensitivity and aberrations of eyes after implantation of one of the MIOLs mentioned above. Through comparison, we focused on the efficacy, accuracy, predictability, safety and visual quality of the two MIOLs implanted in eyes with axial myopia.

## Methods

### Patients

This was a prospective cohort study. Cataract patients who underwent cataract surgery with AT LISA tri 839MP or SBL-3 implantation from September 2017 to January 2020 at Department of Ophthalmology, Peking University Third Hospital were enrolled in this study. Informed consent was obtained from patients before data collection. Patients decided which IOL was to be implanted. For patients with bilateral MIOL implantation, only one randomly selected eye was to be included in this study. This study was conducted in accordance with the Declaration of Helsinki and received the approval of Peking University Third Hospital Science Research Ethics Committee (IRB00006761-M2019414). The study was retrospectively registered and posted on clinicaltrials.gov at 12/02/2020 (NCT04265846).

The inclusion criteria were as followed: (1) age of patients more than 18 years old; (2) eyes with axial length (AL) ranging from 22.00 mm to 28.00 mm; (3) eyes with prediction of postoperative corneal astigmatism less than 1.0 D; (4) eyes with photopic pupil diameter ranging from 2.75 mm to 5.75 mm; (5) eyes with angle kappa less than 0.5 mm; (6) eyes with corneal spherical aberration less than 0.5 μm. Exclusion criteria were serious intraoperative complications, glaucoma, pseudoexfoliation syndrome, uveitis, macular degeneration or other retinal impairment, corneal scarring, amblyopia or having difficulties with examinations or 3 months’ follow-up.

According to whether the AL more than 24.00 mm and the IOL implanted, eyes were divided into four groups. They are the axial myopia trifocal group (Group A), the control trifocal group (Group B), the axial myopia bifocal group (Group C) and the control bifocal group (Group D).

### Intraocular lenses

The AT LISA tri 839MP IOL is a diffractive trifocal IOL with a diffractive profile on its anterior surface. It is a bi-aspheric IOL with a -0.18 spherical aberration. It is made of foldable hydrophilic acrylic material with a water content of 25%, while its surface is hydrophobic. This preloaded IOL has a 6.0 mm biconvex optic, a total diameter of 11.0 mm and a 4-haptic design. Its edge is designed to be 360-degree square to prevent posterior capsule opacification. In addition, there is a trifocal diffractive pattern within a diameter of 4.3 mm and bifocal pattern between 4.3 mm and 6.0 mm of diameter. The near add of the IOL is + 3.33 D, while the intermediate add is + 1.66 D. The available spherical power is between 0.00 and + 32.00 D with 0.50D increments [3, 21].

As a refractive rotationally asymmetric bifocal IOL, the SBL-3 IOL is a bi-aspheric hydrophilic acrylic IOL with a neutral aberration profile, a 5.75 mm optic and an 11.0 mm total diameter. It has a near segment with a + 3.00 D addition in the inferior anterior optic. The near segment occupies 42% of the optic and the distance segment occupies 50%. A small wedge-shaped transition zone separates them. The available spherical power is between + 10.00 and + 36.00D with 0.50D increments, with the most commonly used powers (range from + 15.00D to + 25.00D) being available in 0.25D increments [7].

### Preoperative examinations

All patients underwent full preoperative examinations, including slitlamp evaluation, tonometry, manifest refraction, biometric evaluation (IOLMaster 500, Carl Zeiss Meditec AG), corneal aberrometry and topography (Pentacam HR, Oculus Optikgerate GmbH), dilated fundoscopy and retinal optical coherence tomography examination (Cirrus 4000, Carl Zeiss Meditec AG). Uncorrected and corrected distance visual acuity were measured at 5 m with logMAR E chart. Visual acuity was recorded in the form of logarithm of the minimum angle of resolution (logMAR) value.

### IOL power calculation and refractive target strategy

Holladay 2 formula was applied to calculate the IOL power for all eyes (IOLMaster 500, Carl Zeiss Meditec AG). Optimized A-constants of the trifocal IOL and the bifocal IOL for the surgeon in this study were applied. To improve the intermediate visual acuity and avoid distant drift of the near focal point and hyperopia and presbyopia symptoms, a mild myopic target SE was expected for every group [22]. However, as IOL type had significant influence in the accuracy of IOL power calculation, personalized refractive targets were selected based on clinical experience (a target of -0.10 D for the trifocal IOL and 0.10 D for the bifocal IOL).

### Surgical technique

All surgical procedures were performed by the same experienced surgeon (HQ) using topical anesthesia. If the corneal astigmatism of the surgery eye was lower than 0.50 D, a primary incision located at 135° and an auxiliary incision located at 45° were created, or an incision on the preoperative steep meridian of the corneal astigmatism was chosen. The incisions were all created under the conduction of Callisto Eye System (Carl Zeiss Meditec). After a 5.0–5.5 mm anterior capsulorhexis was created and phacoemulsification (Centurion Vision System, Alcon Laboratories Inc), an AT LISA tri 839MP IOL or an SBL-3 IOL was implanted into the capsular bag. All patients followed the same postoperative regimen for 1 month, including 1 drop each of levofloxacin, diclofenac sodium and prednisolone acetate 4 times a day. The frequency decreased by 1 time a week.

### Postoperative examinations

Patients underwent routine examinations, including visual acuity, tonometry and slitlamp evaluation at 1 day, 1 week and 1 month after surgery. Besides, all patients underwent comprehensive evaluation 3 months postoperatively. Examinations included manifest refraction, uncorrected and distance-corrected visual acuity at far, intermediate (80 cm) and near (40 cm) distances (UDVA, UIVA, UNVA, CDVA, CIVA and CNVA), contrast sensitivity and aberrations. Using the OPTEC 6500 Vision Tester (Stereo Optical Co. Inc, Chicago, USA), contrast sensitivity was conducted under four conditions, including photopic(85 cd/m^2^), mesopic(3 cd/m^2^), photopic with glare and mesopic with glare. Besides, there are five spatial frequencies (1.5, 3, 6, 12, and 18 cycles per degree [cpd]) under every condition. Aberrations were measured with OPD-Scan III (NIDEK Technologies, Japan). A certified optometrist who was independent of the surgeons and main investigators performed all the postoperative examinations.

### Statistical analysis

Statistical analysis was performed using SPSS Statistics for Windows software (version 22.0, IBM Corp, USA). Kolmogorov–Smirnov test was used to assess the consistency between the sample and normal distribution. If the variants are in accordance with normal distribution, one-way ANOVA was used to compare the mean between groups. Otherwise, Kruskal–Wallis H test was applied. Researchers performing statistical analysis were also blinded. For all statistical analysis, data were expressed as mean ± SD. A *P* value less than 0.05 was considered statistically significant.

## Results

### Demographic and preoperative parameters

A total of 80 eyes of 80 patients (36 males and 44 females) were included in this study, with 20 eyes in Group A (7 males and 13 females), 20 eyes in Group B (11 males and 9 females), 20 eyes in Group C (14 males and 6 females) and 20 eyes in Group D (4 males and 16 females). The average age of all patients was 68.9 ± 10.6 years old. As shown in Table 1, there was no significant difference in age, uncorrected and corrected distance visual acuity and corneal astigmatism among four groups (*P* = 0.455, 0.423, 0.655 and 0.735). No significant difference of AL, anterior chamber depth, pupil diameter, preoperative SE and IOL power were found between two axial myopia groups (*P* = 0.293, 0.697, 0.448, 0.487 and 0.548) or two control groups (*P* = 0.489, 0.836, 0.876, 0.155 and 0.072). There were significant differences of AL, anterior chamber depth and IOL power between the two AT LISA tri 839MP groups (*P* = 0.007, 0.030 and < 0.001) and the two SBL-3 groups (*P* = 0.033, 0.004 and < 0.001). No significant difference of target SE was found between two AT LISA tri 839MP groups (*P* = 0.687) or two SBL-3 groups (*P* = 0.490). However, target SE was significantly different between two axial myopia groups (*P* < 0.001) or two control groups (*P* < 0.001), which meant the selection of target SE of two IOLs was different.

**Table 1:** Demographic and preoperative characteristics

**Parameter**	**AT LISA tri 839MP Groups**		**SBL-3 Groups**		***P* Value**
	**Group A** **Axial Myopia Group**	**Group B** **Control Group**	**Group C** **Axial Myopia Group**	**Group D** **Control Group**	
Age (years)	66.1 ± 13.0	71.0 ± 10.5	70.4 ± 10.3	68.2 ± 8.3	0.455
UDVA (logMAR)	0.41 ± 0.25	0.57 ± 0.34	0.52 ± 0.30	0.45 ± 0.28	0.423
CDVA (logMAR)	0.31 ± 0.23	0.32 ± 0.32	0.37 ± 0.22	0.40 ± 0.36	0.655
SE (D)	-3.86 ± 3.39^b,d^	-0.87 ± 2.14^a^	-3.21 ± 2.51^d^	-0.30 ± 2.64^a,^^c^	< 0.001^*^
Corneal Astigmatism (D)	0.73 ± 0.21	0.72 ± 0.32	0.66 ± 0.33	0.64 ± 0.30	0.735
Pupil diameter (mm)	3.31 ± 0.57^b^	2.79 ± 0.47^a^	3.27 ± 0.77	2.94 ± 0.43	0.035^*^
AL (mm)	25.29 ± 0.84^b^^,d^	23.28 ± 0.36^a^^,c^	25.04 ± 0.64^b,^^d^	23.19 ± 0.41^a,^^c^	< 0.001^*^
ACD (mm)	3.25 ± 0.38^b^^,d^	2.93 ± 0.33^a^^,c^	3.21 ± 0.37^b,^^d^	2.95 ± 0.40^a,^^c^	0.009^*^
IOL power (D)	15.90 ± 3.11^b^^,d^	21.23 ± 0.77^a^^,c^	15.34 ± 2.74^b,^^d^	20.40 ± 1.55^a,^^c^	< 0.001^*^
Target SE (D)	-0.15 ± 0.12^c^^,d^	-0.13 ± 0.19^c,^^d^	0.12 ± 0.07^a^^,b^	0.11 ± 0.06^a,^^b^	< 0.001^*^

One-way ANOVA test or Kruskal–Wallis H test; All data are expressed as mean ± SD

*UDVA* Uncorrected distance visual acuity, *CDVA* Corrected distance visual acuity, *logMAR* Logarithm of the minimum angle of resolution, *SE* Spherical equivalent, *D* Dioptre, *AL* Axial length, *ACD* Anterior chamber depth, *IOL* Intraocular lens

^a^*P* < 0.05 versus Group A (the axial myopia trifocal group)

^b^*P* < 0.05 versus Group B (the control trifocal group)

^c^*P* < 0.05 versus Group C (the axial myopia bifocal group)

^d^*P* < 0.05 versus Group D (the control bifocal group)

^*^P < 0.05 among four groups

### The efficacy after implantation of the two IOLs

**Fig. 1:**
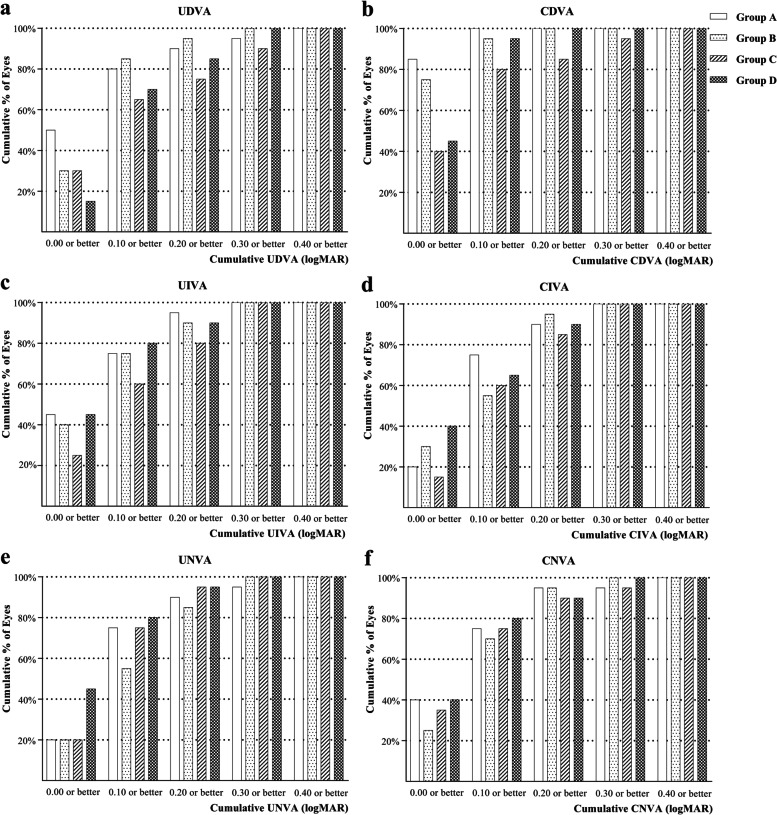
The distribution of cumulative visual acuity of four groups three months after implantation. (CDVA = corrected distance visual acuity; CIVA = corrected intermediate visual acuity; CNVA = corrected near visual acuity; logMAR = logarithm of the minimum angle of resolution; UDVA = uncorrected distance visual acuity; UIVA = uncorrected intermediate visual acuity; UNVA = uncorrected near visual acuity)

Figure 1 showed the distribution of cumulative visual acuity of four groups three months after implantation. As is shown in Table 2, only the CDVA of Group A and B was significantly better than Group C (*P* = 0.007 and 0.043). It is consistent with the percentages of eyes reached 0.00 logMAR shown in Fig. 1b. No other significant difference was found in uncorrected and distance-corrected visual acuity at different distances (all *P* > 0.05).

**Table 2:** Visual acuity and refractive results three months after implantation

**Parameter**	**AT LISA tri 839MP Groups**		**SBL-3 Groups**		***P* Value**
	**Group A** **Axial Myopia Group**	**Group B** **Control Group**	**Group C** **Axial Myopia Group**	**Group D** **Control Group**	
UDVA (logMAR)	0.07 ± 0.10	0.06 ± 0.07	0.13 ± 0.17	0.09 ± 0.07	0.315
UIVA (logMAR)	0.08 ± 0.09	0.09 ± 0.11	0.13 ± 0.10	0.08 ± 0.10	0.377
UNVA (logMAR)	0.12 ± 0.10	0.14 ± 0.11	0.11 ± 0.08	0.08 ± 0.09	0.234
CDVA (logMAR)	0.01 ± 0.02^c^	0.02 ± 0.04^c^	0.09 ± 0.14^a^^,b^	0.04 ± 0.04	0.003^*^
CIVA (logMAR)	0.11 ± 0.09	0.12 ± 0.10	0.14 ± 0.09	0.10 ± 0.10	0.669
CNVA (logMAR)	0.09 ± 0.10	0.11 ± 0.09	0.10 ± 0.11	0.09 ± 0.10	0.802
Sphere (D)	0.11 ± 0.38	-0.11 ± 0.46	-0.03 ± 0.39	-0.15 ± 0.42	0.188
Cylinder (D)	-0.75 ± 0.41	-0.56 ± 0.44	-0.51 ± 0.50	-0.54 ± 0.53	0.337
SE (D)	-0.26 ± 0.32	-0.39 ± 0.36	-0.28 ± 0.40	-0.42 ± 0.45	0.478
Prediction error of SE (D)	0.11 ± 0.34^c^^,d^	0.26 ± 0.33^d^	0.40 ± 0.37^a^	0.52 ± 0.44^a,^^b^	0.005^*^

Kruskal-Walli one-way ANOVA test; All data are expressed as mean ± SD

*UDVA* Uncorrected distance visual acuity, *UIVA* Uncorrected intermediate visual acuity, *UNVA* Uncorrected near visual acuity, *CDVA* Corrected distance visual acuity, *CIVA* Corrected intermediate visual acuity, *CNVA* Corrected near visual acuity, *logMAR* Logarithm of the minimum angle of resolution, *D* diopter, *SE* Spherical equivalent

^a^*P* < 0.05 versus Group A (the axial myopia trifocal group)

^b^*P* < 0.05 versus Group B (the control trifocal group)

^c^*P* < 0.05 versus Group C (the axial myopia bifocal group)

^d^*P* < 0.05 versus Group D (the control bifocal group)

^*^P < 0.05 among four groups

### The accuracy after implantation of two IOLs

**Fig. 2:**
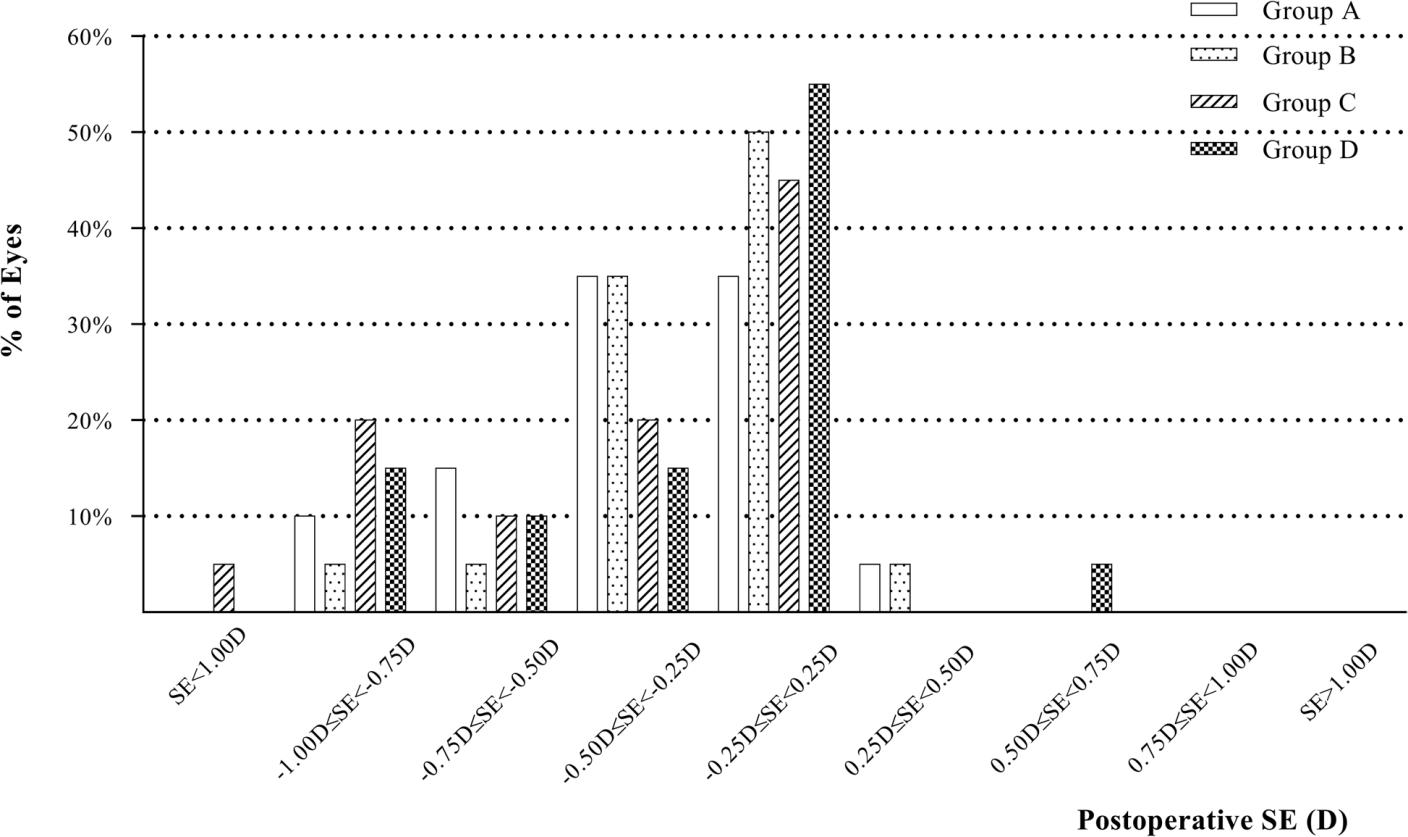
The SE refractive accuracy of four groups three months after implantation. (D = dioptre; SE = spherical equivalent)

The difference between postoperative SE and emmetropia was used to analyze the accuracy. The percentages of eyes with postoperative SE between -0.25D and 0.25D in four groups were 35%, 50%, 45% and 55% (Fig. 2). Most of eyes in four groups had postoperative SE ranged from -1.00D to 0.25D. There was no significant of postoperative SE among four groups (Table 2, *P* = 0.478).

### The predictability after implantation of two IOLs

**Fig. 3:**
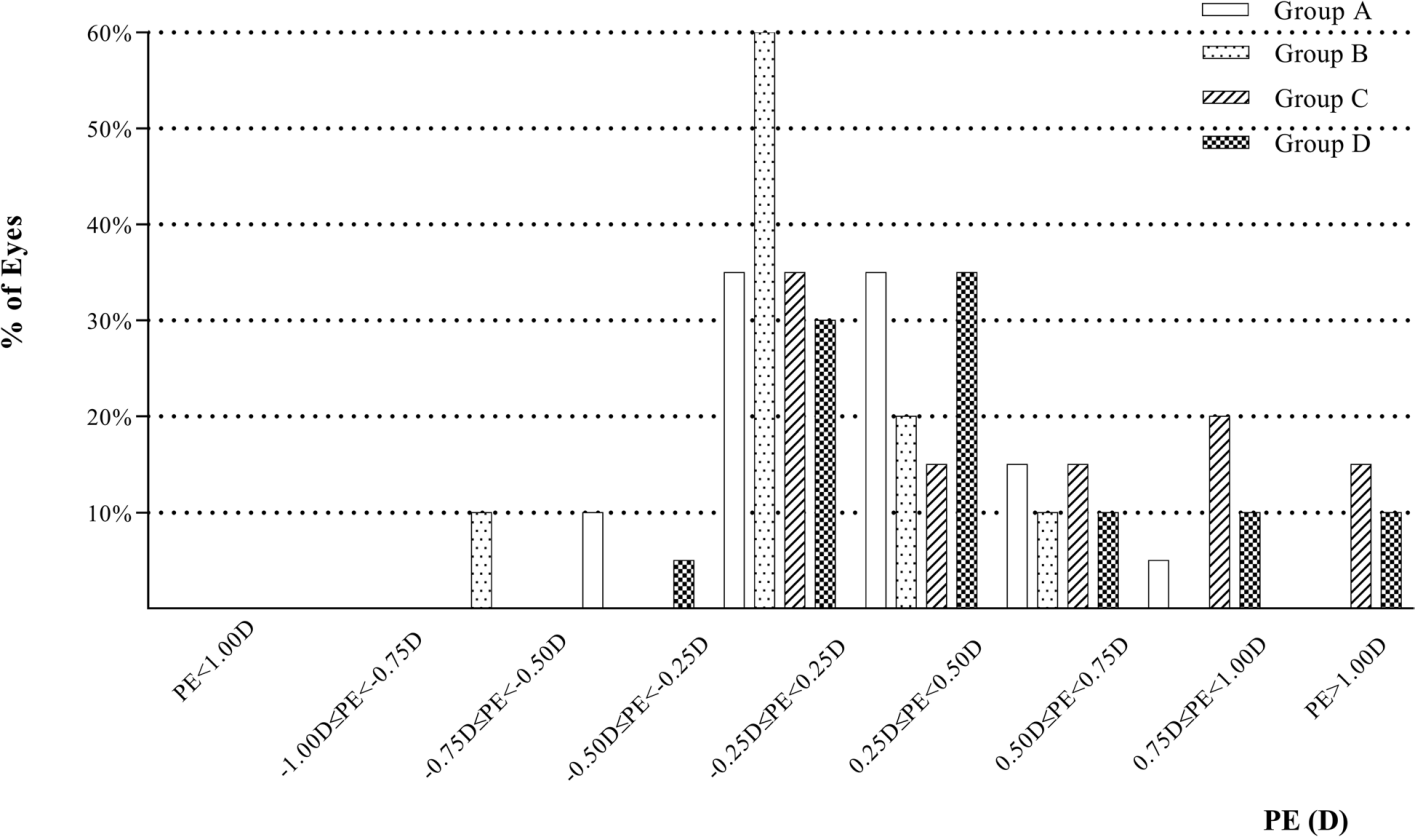
The PE of postoperative SE of four groups three months after implantation. (D = dioptre; PE = prediction error; SE = spherical equivalent)

The prediction error was the difference between target SE and postoperative SE. The prediction error of two AT LISA tri 839MP groups mostly ranged from -0.25D to 0.75D, while that of two SBL-3 groups mostly ranged from -0.25D to 1.00D or more (Fig. 3). As is shown in Table 2, there was no difference of prediction error between two AT LISA tri 839MP groups or two SBL-3 groups (*P* = 0.202 and 0.300). However, Group A had a smaller prediction error than Group C (*P* = 0.015), and Group B had a smaller prediction error than Group D (*P* = 0.027).

### The safety after implantation of two IOLs

During the three months’ follow-up, the CDVA of all eyes was better than that preoperatively. No postoperative complication was found. All patients will take a longer follow-up to confirm the long-term safety and stability.

### Contrast sensitivity

**Fig. 4:**
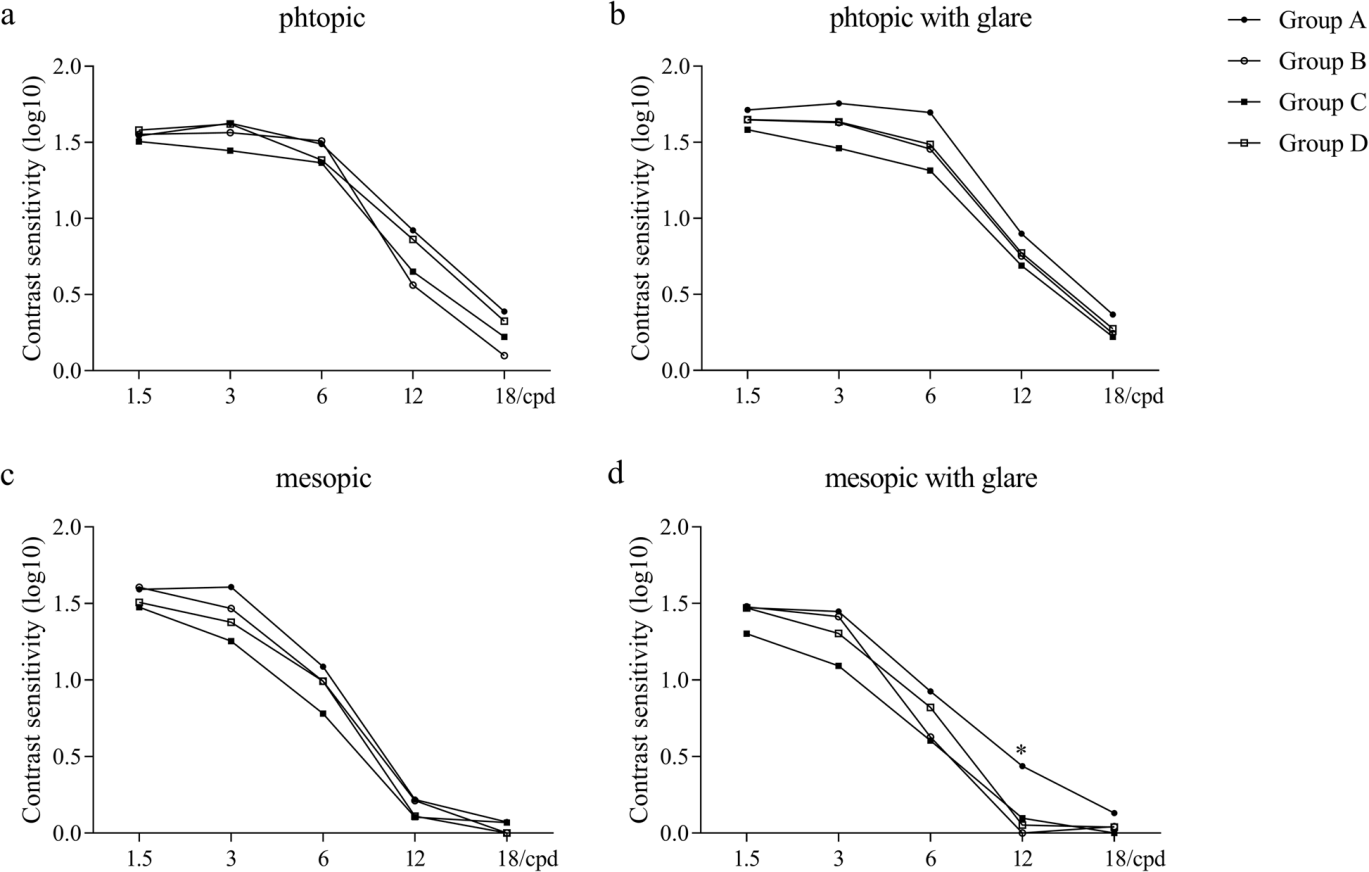
Contrast sensitivity curves of four groups three months after implantation. (cpd = cycles per degree; *Statistically significant difference [*P* < 0.05])

The graphs of mean contrast sensitivity at five different frequencies under different light conditions were shown in Fig. 4. No significant difference of contrast sensitivity was found among four groups at any spatial frequency and under any light condition, except the contrast sensitivity of Group A was better than the other three groups at 12 cpd under mesopic with glare condition (*P* = 0.008, 0.049 and 0.014).

### Aberrations

There was significant difference of total aberration, higher order aberration, trefoil aberration and modulation transfer function (MTF) between two axial myopia groups (Table 3, all *P* < 0.001) or two control groups (all *P* < 0.001). The coma aberration of Group A was significantly smaller than Group C (*P* = 0.021), and that of Group B also had a trend to be smaller than Group D (*P* = 0.056). As for spherical aberration, significant difference was only found between Group A and Group D (*P* = 0.048). The Strehl ratio (SR) of Group A and B were higher than that of Group C and D (*P* < 0.001).

**Table 3:** Aberrations, MTF and SR three months after implantation

**Parameter**	**AT LISA tri 839MP Groups**		**SBL-3 Groups**		***P* Value**
	**Group A** **Axial Myopia Group**	**Group B** **Control Group**	**Group C** **Axial Myopia Group**	**Group D** **Control Group**	
Total aberration	0.59 ± 0.22^c,d^	0.68 ± 0.21^c,d^	1.24 ± 0.14^a,b^	1.24 ± 0.19^a,b^	< 0.001^*^
Higher order aberration	0.26 ± 0.12^c,d^	0.30 ± 0.12^c,d^	0.72 ± 0.13^a,b^	0.75 ± 0.16^a,b^	< 0.001^*^
Coma aberration	0.07 ± 0.05^c,d^	0.09 ± 0.06	0.15 ± 0.06^a^	0.16 ± 0.07^a^	0.001^*^
Spherical aberration	0.01 ± 0.01^d^	0.02 ± 0.02	0.03 ± 0.02	0.04 ± 0.02^a^	0.022^*^
Trefoil aberration	0.23 ± 0.12^c,d^	0.25 ± 0.13^c,d^	0.68 ± 0.14^a,b^	0.70 ± 0.18^a,b^	< 0.001^*^
MTF	36.58 ± 10.18^c,d^	34.05 ± 9.29^c,d^	22.07 ± 2.81^a,b^	21.66 ± 2.82^a,b^	< 0.001^*^
SR	0.05 ± 0.03^c,d^	0.04 ± 0.03 ^c,d^	0.02 ± 0.01^a,b^	0.02 ± 0.01 ^a,b^	< 0.001^*^

Kruskal-Walli one-way ANOVA test; All data are expressed as mean ± SD

*MTF* Modulation Transfer Function, *SR* Strehl ratio

^a^*P* < 0.05 versus Group A (the axial myopia trifocal group)

^b^*P* < 0.05 versus Group B (the control trifocal group)

^c^*P* < 0.05 versus Group C (the axial myopia bifocal group)

^d^*P* < 0.05 versus Group D (the control bifocal group)

^*^P < 0.05 among four groups

## Discussion

AL will affect the visual quality after IOL implantation in many ways. Many ophthalmologists are cautious about the use of MIOL in patients with axial myopia. As cataract formation would increase the degree of myopia, AL was an important parameter to evaluate the refractive status of cataract patients. According to a large sample study in 2018, the average of AL was 23.96 mm of 13 301 cataract eyes and 23.89 mm of another 5 200 cataract eyes [18]. Another population‐based study included 2 957 adults with an average AL of 23.67 mm [23]. And there were several studies selected 24.00 mm as the inclusion criterion for myopia patients [24, 25]. In addition, several studies have concluded that AT LISA tri 839MP provided satisfactory short-term visual outcomes in highly myopic eyes with low IOL power (0.00 to 10.00 D) [19], but its retinal concerns in the long-term follow-up were also very important [26]. The available spherical power of SBL-3 was between + 10.00 and + 36.00 diopter (D) [7]. The lowest power of + 10.00 D corresponded to an AL of around 26.00 mm. It could not be applied in eyes with higher AL. However, the most commonly used powers (range from + 15.00 to + 25.00 D) of SBL-3 was available in 0.25 D increments, which provided more accurate powers than other multifocal IOLs. Along with its excellent performance in the intermediate visual acuity and reasonable price, it was very meaningful to study the application of SBL-3 in patients with mild and moderate myopia [7]. To sum up, the AL over 24.00 mm was considered as a grouping criterion to evaluate the efficacy, accuracy, predictability, safety and visual quality of the two MIOLs in eyes with axial myopia.

The result of demographics showed similar characteristics, which made the data of eyes in four groups can be compared with less statistical bias. During the three months’ follow-up, the CDVA of all eyes was better than that preoperatively. Three months after implantation, we found satisfactory visual results in the two axial myopia groups. The postoperative visual acuity at far, intermediate and near distances in four groups are similar to findings in previous studies with the same length of postoperative follow-up [9, 19, 27]. Wang, et al. had concluded that SBL-3 IOL provided a relatively wider range of intermediate vision in bifocal IOLs [28]. Consistent with that, the UIVA and CIVA of SBL-3 IOL was similar to the trifocal IOL in this study. These all indicated that both MIOLs provided reliable effects for eyes with axial myopia.

In previous studies, long AL has been proved to strongly influence the accuracy of IOL power prediction [15, 16]. The prediction errors are partially because of measurement errors. To avoid this, IOLMaster 500, a partial coherence interferometry measurement, was used to improve the accuracy of preoperative biometric evaluation in this study [29]. In addition, the limited performance of third-generation formulas also accounted for the prediction errors [30, 31]. Cheng et al. applied modified AL adjustment and successfully improved the accuracy [32]. In this study, we used Holliday 2 formula, a fourth-generation formula that takes into consideration more variables, to calculate IOL power and predict residual SE. It showed relatively high accuracy for AL ranged from 21.00 to 27.00 mm [18]. Relatively new formulas, such as Barrett Universal II formula and Olsen formula, did have higher accuracy in IOL power calculation across a wide range of AL [18]. But they were not available in China mainland when the study started. With the optimized A-constants of the trifocal IOL and the bifocal IOL and personalized refractive target strategy, all the four groups in this study achieved similar mild myopic SE postoperatively, which was consistent with the expectation.

According to the analysis of the correlation between prediction error and AL in a study which included 13 301 eyes with a monofocal IOL in America, Holladay 2 formula produced myopic drift outcomes for eyes with AL shorter than 24.00 mm, and hyperopic drift outcomes for eyes with AL longer than 24.00 mm [18]. However, Chinese eyes have been shown to have smaller anterior chamber depth, which influenced the IOL power calculation a lot [33]. And the A-constants of different IOLs also had a big impact. These would have a significant impact on IOL power calculation. In this study, there was a tendency that axial myopia groups had smaller degree of myopia drift than corresponding control group. It was consistent with the pattern mentioned above and would be more obvious if more patients with longer AL were included. Besides, the predictability of AT LISA tri 839MP IOL was better than that of SBL-3 when applying the Holladay 2 formula, whether in axial myopia groups or control groups. To sum up, both AL and IOL type played very important roles in personalized MIOL application in eyes with axial myopia, but the influence of AL was not so obvious in the AL range in this study.

Contrast sensitivity describes the ability of the visual system to discern between luminances of different levels in a static image. Contrast sensitivity and visual acuity describe different aspects of vision. In this study, the contrast sensitivity of four groups was similar at any spatial frequency and under any light condition, except the trifocal group with axial myopia had a better performance at 12 cpd under mesopic with glare condition. In view of the similar results of contrast sensitivity of the two MIOLs under day condition, the difference under other conditions may come from different IOL designs. There is a trifocal diffractive pattern within a diameter of 4.3 mm and bifocal pattern between 4.3 mm and 6.0 mm of diameter at the optic of AT LISA tri 839MP. In dark environment, the pupils of a patient will enlarge so that more light will pass the bifocal area. It will increase the proportion of light energy allocated to the far focus, which will improve distance visual acuity. This can account for the better performance of AT LISA tri 839MP under night or night with glare condition to some extent. Further studies are needed to thoroughly explain the difference of contrast sensitivity in this study.

The optical characteristics of the IOL itself play an important role in postoperative aberrations. The two IOLs both have a bi-aspheric design. AT LISA tri 839MP IOL is a diffractive trifocal IOL with a -0.18 spherical aberration [3, 21]. SBL-3 IOL is a refractive rotationally asymmetric bifocal IOL with a neutral aberration profile. Data analysis shows that the spherical aberrations of four groups were similar, which indicated that the bi-aspheric design had a good performance in the reduction of spherical aberration [34]. However, the higher order aberration, coma aberration, trefoil aberration and MTF of AT LISA tri 839MP IOL were better than those of SBL-3 IOL. Correspondingly, the Strehl ratio of AT LISA tri 839MP IOL was higher. Montes-Mico R et al. also concluded that the diffractive IOL provided better optical quality than the rotationally asymmetric IOL [35]. The diffractive IOL had higher MTF values at all spatial frequencies compared with the rotationally asymmetric IOL. MTF shows how an optical system transmits spatial frequencies, which is closely related to the details of an object. Higher MTF values mean higher image quality.

As important parts of higher order aberration, coma aberration and trefoil aberration are both closely related to the decentration and tilt of IOL [36–38]. IOL in eyes with a long AL tended to have a higher risk of decentration and tilt [39, 40]. Decentration and tilt had a significant impact on optical quality with IOLs, being more severe with the rotationally asymmetric IOL [35]. Thereby, the visual acuity may also be influenced. It is consistent with the result of CDVA in this study. In addition, the postoperative rotation, decentration and tilt of IOL are also important problems for long AL eyes. Bert et al. reported a moderately myopic patient whose AT LISA tri toric 939MP IOL (Carl Zeiss Meditec AG), an undersized plate-haptic trifocal toric IOL, had rotated and tilted due to insufficient fixation in the large capsular bag of the myopic eye [41]. Further studies about IOLs in highly myopic eyes are needed to better clarify the mechanism.

Besides decentration and tilt, the rotation of IOL will influence the postoperative vision, especially for eyes with SBL-3. The rotation of SBL-3 will lead to the unbalanced distribution of light energy between the distance segment and the near segment [42, 43]. A long-term postoperative follow-up is necessary for myopic patients to detect postoperative complications in time, including the rotation and decentration of IOL.

The long-term efficacy and stability of IOLs are very important. It was a limitation of this study that there was no long-term assessment. Jaime et al. have conducted a 2-year assessment after AT LISA tri 839MP implantation in high myopic patients and concluded that retinal concerns, such as retinal detachment, could not be ignored in the long-term development [26]. A second limitation is the relatively small sample size, which was as a result of the strict inclusion criteria. As myopia is closely related to other conditions affecting visual acuity such as myopic maculopathy [44], the implantation of MIOLs for myopic patients must be very cautious. The risk of surgery complications, such as IOL decentration, is also higher. Detailed preoperative evaluation was also the basis of this study. A third limitation was the relatively small range of AL in this study, which was mainly limited by the IOL power range of SBL-3. Besides, binocular visual outcomes, which could reflect real world vision, were not included in this study. Many binocular parameters, including binocular vision, life quality evaluation, overall satisfaction and so on, also played important roles after IOL implantation.

We can conclude that the diffractive trifocal IOL and the refractive rotationally asymmetric bifocal IOL both provided good efficacy, accuracy, predictability and safety for eyes with axial myopia. By contrast, the diffractive trifocal IOL had a better performance in corrected distance visual acuity and visual quality.

## Data Availability

The datasets generated and analyzed during the current study are not publicly available due to ethical restrictions but are available from the corresponding author on reasonable request. The data that support the findings of this study are available from the corresponding author, Hong Qi, upon reasonable request.

## Abbreviations

ACD: Anterior chamber depth
AL: Axial length
CDVA: Corrected distance visual acuity
CIVA: Corrected intermediate visual acuity
CNVA: Corrected near visual acuity
cpd: Cycles per degree
D: Dioptre
IOL: Intraocular lens
logMAR: Logarithm of the minimum angle of resolution
MIOL: Multifocal intraocular lens
MTF: Modulation Transfer Function
PE: Prediction error
SE: Spherical equivalent
SR: Strehl ratio
UDVA: Uncorrected distance visual acuity
UIVA: Uncorrected intermediate visual acuity
UNVA: Uncorrected near visual acuity
